# The Xpert MTB/RIF Ultra assay detects *Mycobacterium tuberculosis* complex DNA in white rhinoceros (*Ceratotherium simum*) and African elephants (*Loxodonta africana*)

**DOI:** 10.1038/s41598-020-71568-9

**Published:** 2020-09-02

**Authors:** Wynand J. Goosen, Tanya J. Kerr, Léanie Kleynhans, Robin M. Warren, Paul D. van Helden, David H. Persing, Sven D. C. Parsons, Peter Buss, Michele A. Miller

**Affiliations:** 1 DSI-NRF Centre of Excellence for Biomedical Tuberculosis Research, South African Medical Research Council Centre for Tuberculosis Research, Division of Molecular Biology and Human Genetics, Faculty of Medicine and Health Sciences, Stellenbosch University, PO Box 241, Cape Town, 8000 South Africa; 2 Cepheid, Sunnyvale, CA USA; 3 Veterinary Wildlife Services, Kruger National Park, South African National Parks, Skukuza, South Africa

**Keywords:** Molecular biology, Diseases, Medical research, Molecular medicine

## Abstract

The study describes the novel use of the Xpert MTB/RIF Ultra assay for detection of *Mycobacterium tuberculosis* complex (MTBC) DNA in samples from white rhinoceros (*Ceratotherium simum*) and African elephants (*Loxodonta africana*). Culture negative respiratory sample matrices were spiked to determine if the Ultra could detect MTBC DNA in rhinoceros and elephant samples. Rhinoceros bronchial alveolar lavage fluid (BALF) was found to have an inhibitory effect on the Ultra. In this study, the limit of detection (LOD) of *M. tuberculosis* H37Rv in all spiked animal samples were 2 CFU/ml compared to 15.6 CFU/ml for humans, while the LOD for *M. bovis* SB0121 was 30 CFU/ml compared to 143.4 CFU/ml for *M. bovis* BCG in humans. Screening was performed on stored tissue and respiratory samples from known MTBC-infected animals and MTBC DNA was detected in 92% of samples collected from six rhinoceros and two elephants. Conversely, 83% of culture-negative tissue and respiratory samples from uninfected animals tested negative on the Ultra. In conclusion, the Ultra assay appears to be a sensitive and rapid diagnostic test for the detection of MTBC DNA from tissue and respiratory samples collected from African elephants and rhinoceros. Furthermore, the Ultra assay could provide a new tool for the detection of MTBC in various sample types from other wildlife species.

## Introduction

*Mycobacterium tuberculosis* complex (MTBC) members, *Mycobacterium bovis* (*M. bovis*) and *Mycobacterium tuberculosis* (*M. tuberculosis*), are the cause of bovine tuberculosis (bTB) and human tuberculosis (TB), respectively. These chronic infectious diseases are a significant global health threat for human and animal populations. Since many high TB burden countries are dependent on animal-related industries such as agriculture and tourism, the consequences of infection can be devastating to their economies. Increased opportunities for disease transmission at human–animal interfaces occur as human settlements encroach on agricultural lands or natural habitats. Since TB can cross species barriers, cases of bTB in people in African countries have been reported^1,2^ as well as reports of *M. tuberculosis* infection in domestic cattle in rural areas of Africa as well as captive wildlife and pets^3–6^.

The risks of MTBC spread to endangered wildlife have been highlighted by the recent discoveries of *M. bovis* infection in free-ranging white and black rhinoceros’ (*Ceratotherium simum, Diceros bicornis*) as well as the death of an African elephant (*Loxodonta africana*) bull shown to have *M. tuberculosis* infection in the Kruger National Park (KNP)^4,5,7^. Infection of these animals was not unexpected since KNP is endemic for bTB; however, *M. tuberculosis* infection in a free-ranging elephant is a novel finding. As controlled veterinary diseases, infections with MTBC have significant consequences for species management, public health, veterinary disease control, and conservation endeavours.

Due to the paucity of diagnostic tests and disease surveillance programs for wildlife, MTBC infections can remain undetected for years, resulting in uncontrolled spread^8^. In livestock, *M. bovis*-exposure within a population is often indirectly detected through the antemortem measurement of the host’s antigen-specific cell-mediated immune (CMI) responses, rather than directly detecting the presence of the pathogen to determine the presence of an active infection and shedding by the host. Using a CMI-based test is a valuable tool for surveillance to determine the presence of exposure before decisions on mitigation are undertaken. However, in vitro diagnostic assays for detection of host CMI responses to MTBC are either very limited, as in the case of African rhinoceros^9^, or do not yet exist for African elephants. Therefore, antemortem TB diagnosis in these species still relies on mycobacterial culture of respiratory tract samples such as bronchoalveolar lavage fluid (BALF) from rhinoceros or BALF and trunk washes (TW) from African elephants.

Mycobacterial culture for wildlife testing is regarded as an imperfect standard^10,11^, perhaps due to aggressive decontamination methods applied to polymicrobial field samples, and results take weeks to months to be delivered. Therefore, detection of mycobacterial DNA in animal samples has been used as an alternative or ancillary diagnostic method^12^. In humans, the Xpert MTB/RIF assay (Cepheid) was initially developed to improve TB and rifampicin resistance (RIF-R) detection using human sputum^13^. To improve performance in paucibacillary samples, a next generation Xpert semi-quantitative assay, the Xpert MTB/RIF Ultra test (Ultra), was developed by improving several features including the first ever combined detection of two genetic insertion elements IS*6110* and IS*1081* for MTBC detection. Notably, field isolates from the MTBC contain varying copies of IS*6110* and IS*1081* between specific strains that may have an influence on a test’s limit of detection (LOD). Varying copy numbers between strains within isolates from the MTBC largely depends on the geographical location of the infected hosts^14,15^. However, the additional detection of IS*1081* by the Ultra, has truly increased its diagnostic appeal for use on specimens collected from animals suspected of having MTBC infections^16^. Initial analytical studies in human sputum have shown the LOD of the Ultra to be 15.6 CFU/ml for *M. tuberculosis* H37Rv and 143.4 CFU/ml for *M. bovis* BCG with a sensitivity of 89% and specificity 98% for *M. tuberculosis* H37Rv detection^16^.

In this study, we describe the first use of the Ultra for the detection of *M. tuberculosis* and *M. bovis* from experimentally spiked animal samples and stored clinical tissue and respiratory tract samples collected from white rhinoceros and African elephants with known MTBC-infection status.

## Results

### Detection range of the Ultra assay in animals

**Figure 1:**
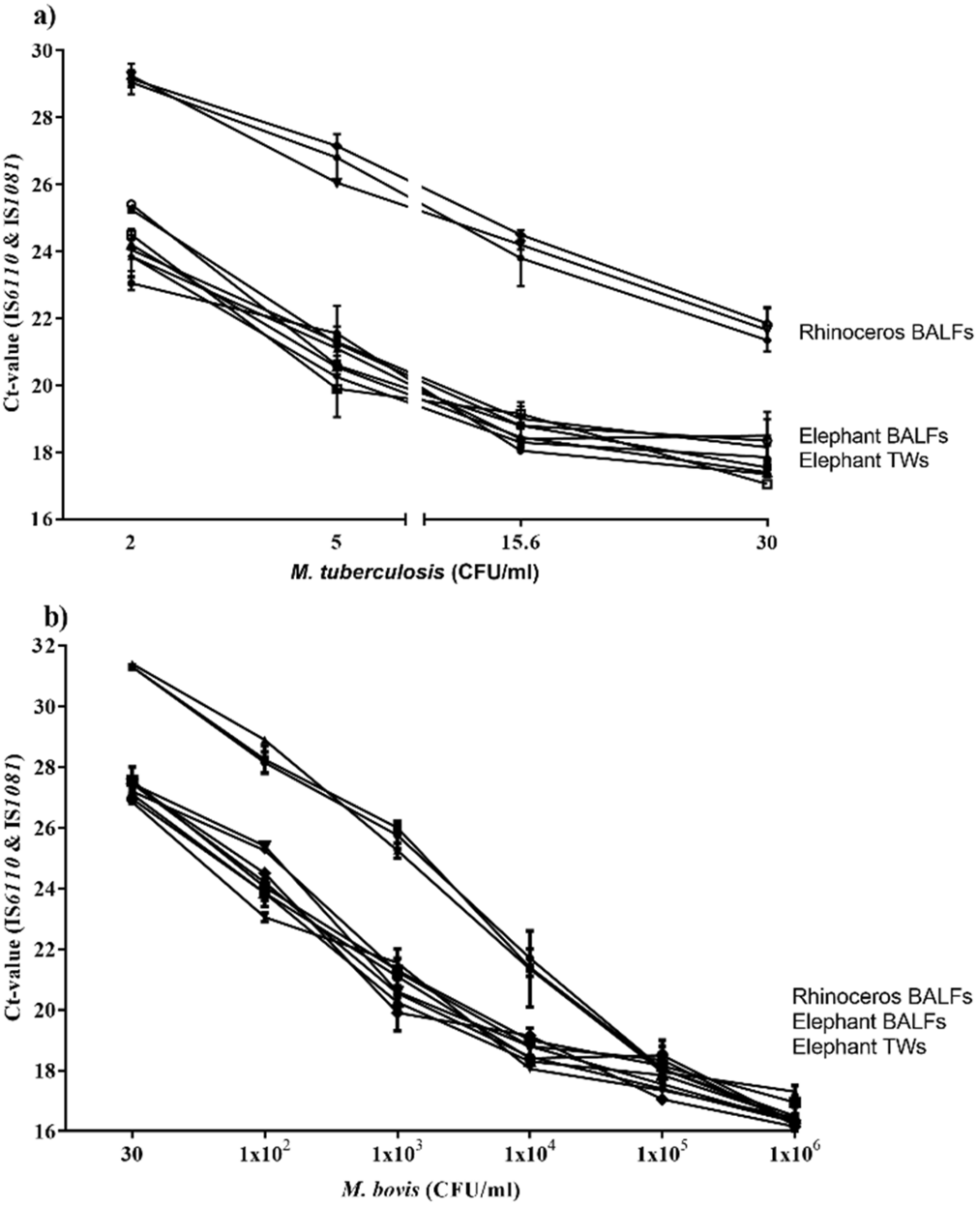
The Xpert MTB/RIF Ultra detection results (represented as mean Ct-values and interquartile ranges) of bronchial alveolar lavage fluids (BALF) and trunk washes (TW) collected from uninfected African elephants (n = 6) (BALF/TW—bottom lines) and rhinoceros (n = 3) (BALF—top three lines), experimentally spiked in duplicate with different concentrations (CFU/ml) of (**a**) *M. tuberculosis* H37Rv (30—2 and 0 CFU/ml) and (**b**) *M. bovis* SB0121 (1 × 10^6^—30 and 0 CFU/ml), respectively. African rhinoceros BALF and African elephant BALF/TW samples were also spiked (in triplicate) with 1 × 10^6^ CFU/ml of *Mycobacterium avium* and ‘MTB NOT DETECTED’ reported for all these samples.

The Ultra assay results for serial dilutions of *M. tuberculosis* and *M. bovis*, spiked into different matrices, are shown in Fig. 1. Briefly, the presence of DNA and RIF susceptibility were detected for all *M. tuberculosis* concentrations (30, 15.6, 5 and 2 CFU/ml), spiked into sets of elephant and rhinoceros respiratory tract samples. The lowest *M. tuberculosis* concentration of 2 CFU/ml was detected in this study (Fig. 1a). In contrast, *M. bovis* spiked respiratory tract samples at similar concentrations were undetectable at concentrations of 15.6 CFU/ml and below (Fig. 1b). However, *M. bovis* spiked samples, at concentrations of 30 CFU/ml and above 1 × 10^2^ to 1 × 10^6^ CFU/ml, were all positively detected by the Ultra assay (Fig. 1b). Notably, no amplification was observed for any of the negative controls (respiratory specimens left unspiked). With a Ct value ≤ 37 considered as positive, Ct-value detection ranges of the Ultra were between 17 and 26 for both elephant BALF and TW and between 22 – 30 for *M. tuberculosis* DNA detection in rhinoceros BALF (Fig. 1a). For *M. bovis* detection the calculated Ct-value detection ranges were between 16 and 27 for both elephant BALF and TW and 16–32 for rhinoceros BALF (Fig. 1b).

### Analytical specificity

The specificity of the primers and probes for MTBC used was demonstrated by spiking respiratory tract samples with a high concentration (1 × 10^6^ CFU/ml) of *M. avium*. No signals were generated from the two *M. tuberculosis*-specific detection probes targeting IS*6110* and IS*1081* genes, with a result “MTB not detected” for all replicates tested (Fig. 1).

### Identification MTBC-infected animals from MTBC endemic areas by mycobacterial culture

Six of the 36 white rhinoceros tested were identified as *M. bovis*-infected, based on mycobacterial growth in culture and PCR (Supplemental Table). Positive *M. bovis* cultures were obtained from 9/23 lymph nodes, 1/1 BALF sample (performed postmortem) and 5/6 lung tissue samples from the six individuals. None of the 31 antemortem BALF samples (animal #32 had a duplicate) collected from the 30 free-ranging rhinoceros were mycobacterial culture positive (Table 1 and Supplemental Table).

**Table 1:** Comparative Xpert MTB/RIF Ultra qPCR assay results for lymph node (LN), lung, bronchial alveolar lavage fluid (BALF) and trunk washes (TW) samples collected from African elephants (*Loxodonta africana*) (n = 22) and white rhinoceros (*Ceratotherium simum*) (n = 36) with known mycobacterial culture results from MTBC endemic areas in South Africa.

Species	Sample type	No of samples	Mycobacterial culture sample results		Ultra Pos^b^/MTBC culture Pos samples	Ultra Neg/MTBC culture Neg samples
			MTBC culture positive (MTBC isolate)^a^	MTBC culture negative		
White rhinoceros^c^	LNs	n = 23	9 (*M. bovis*)	14	8/9	9/14
	Lung	n = 6	5 (*M. bovis*)	1	4/5	1/1
	BALF	n = 32	1 (*M. bovis*)	31	1/1	24/31
African elephants^d^	LNs	n = 5	4 (*M. bovis*)	1	4/4	1/1
	Lung	n = 3	2 (*M. tuberculosis; M. bovis*)	1	2/2	0/1
	BALF	n = 21	2 (*M. tuberculosis*)	19	2/2	19/19
	TW	n = 16	1 (*M. tuberculosis*)	15	1/1	14/15

^a^Confirmed MTBC culture positive: *M. bovis*- and *M. tuberculosis*-positive by mycobacterial culture and strain typing (29).

^b^Xpert MTB/RIF Ultra qPCR (Ultra) assay that simultaneously targets the IS*6110* and IS*1081* insertion element sequences present in the genome of *M. tuberculosis*, *M. bovis*, *M. africanum*, *M. Microti*, *M. canetti*, *M. caprea* and *M. pinnipedii.*

^c^African rhinoceros BALF samples tested were collected from free-ranging animals from an endemic wildlife park.

^d^African elephant PCR positive/ culture positive BALFs, TW and lung sample were collected from one zoo captive elephant confirmed to be infected with *Mycobacterium tuberculosis,* one PCR positive/ culture positive LN samples were collected from one free-ranging *M. bovis* confirmed elephant carcass.

Two of the 22 African elephant samples were infected with MTBC; one with *M. tuberculosis* T2 family and another with *M. bovis* SB0121 (Supplemental Table). The captive elephant infected with *M. tuberculosis* had positive cultures from antemortem samples including one trunk wash and two BALF, and a postmortem lung tissue sample (Table 1). The free-ranging *M. bovis*-infected elephant was only sampled postmortem and *M. bovis* isolated from four lymph node and one lung samples (Table 1 and Supplemental Table). The remaining 20 free-ranging elephants were considered uninfected based on negative mycobacterial cultures from the antemortem collection of BALFs and TWs from 19 individuals, respectively and the postmortem collection of one lung and one lymph node sample at necropsy from one additional individual animal found dead in the field (less than 24 h) (Table 1 and Supplemental Table).

### Ultra’s performance on clinical specimens

Of the six *M. bovis* culture-confirmed white rhinoceros, the Ultra assay successfully identified all six infected animals from one BALF, eight lymph node, and four lung specimens (Table 1). Thirteen out of 30 samples obtained from these six *M. bovis*-infected rhinoceros had test-positive mycobacterium culture and Ultra assay results, and 10/30 samples were negative in both assays (Table 1). However, there were some discordant outcomes. Only for rhinoceros tissue samples, two were mycobacterial culture-positive but Ultra-negative, and five were culture–negative but Ultra-positive (Table 1). Of the 30 culture-negative rhinoceros, antemortem BALF samples from seven animals tested positive in the Ultra assay (Table 1).

Using samples from the *M. tuberculosis* or *M. bovis* culture-confirmed African elephants, the Ultra assay successfully identified both infected animals (Table 1 and Supplemental Table). Unlike the rhinoceros’ specimens, all samples tested (four lymph nodes, two lung tissues, two BALs and one TW) from these two elephants were concordantly positive in the Ultra assay and mycobacterial culture (Table 1). Of the samples tested from the culture-negative elephants, 34/36 samples were also negative on the Ultra assay, with the additional detection of two test-positive samples (one lung and one TW sample) from this culture-negative cohort (Table 1).

Agreement between the Ultra and culture for all specimens was calculated as κ = 0.63, 95%CI 0.48 to 0.79 and a standard error (SE) of 0.08. According to Landis and Kock, this agreement can be considered as “substantial agreement” on their agreement scale^17^.

## Discussion

The original Xpert MTB/RIF assay was one of the most widely used tests for TB and drug-resistance in humans^18^. However, since its endorsement by the World Health Organization (WHO) in 2010, the manufacturers have continued to enhance its diagnostic performance^13,16^, while unknowingly also adapting it into a candidate *M. bovis* diagnostic tool. Improved features are the inclusion of two new PCR targets within the system that target different multicopy genes (IS*6110* and IS*1081*), conversion of assays into fully nested PCRs, and incorporation of a larger PCR reaction tube that doubles the amount of input DNA. These changes subsequently increased the analytical sensitivity of the assay for the detection of *M. tuberculosis* H37Rv from 81.0% to 87.5%, leaving the specificity unchanged at 98.7%^16^. In addition, the increased sensitivity of the Ultra was also observed through the enhanced LOD for other strains like *M. bovis* BCG. The Xpert MTB/RIF assay previously had a LOD for *M. bovis* BCG of 344.1 CFU/ml (95% CI 297.5–434.0) with the Ultra now having a LOD of 143.4 CFU/ml (95% CI 106.2–243.7). These findings suggest an increased sensitivity can be expected for different strains within the MTBC complex^16,19^*.* Findings from this study support those previously reported by Chakravorty *et* al., 2017 by demonstrating that different isolates such as *M. bovis* SB0121, *M. tuberculosis* H37Rv and *M. tuberculosis* T2 family can be detected using the Ultra assay. Furthermore, our preliminary findings suggest that (1) an overall relative sensitivity of 92.0% may be expected when using Ultra on wildlife tissue and respiratory tract samples and (2) lower LODs for *M. tuberculosis* H37Rv and *M. bovis* SB0121 isolates may also be observed for animal respiratory tract samples compared to that of human sputum. However, it must be noted that the latter observation may also be due to varying copy numbers of IS*6110* and IS*1081* between strain isolates from the MTBC and cannot be solely attributed to the performance of the Ultra in various specimen matrices^15^. Furthermore, interpretations of results from the unvalidated use of the Ultra on wildlife specimens must all be done cautiously since such specimens may interfere with results (pers. comm. Cepheid).

The overall observed “substantial agreement” between Ultra and culture on clinical specimens is supportive of Ultra’s diagnostic potential to successfully differentiate between infected and presumed uninfected animals. However, the agreement between these two platforms was clearly affected by discordant results caused by the enhanced overall sensitivity of PCR technology compared to the known sub-optimal performance of mycobacterial culture techniques^10,11^. With regards to described discordant results, we believe Ultra’s failure to detect two *M. bovis* culture positive rhinoceros tissue samples may have been due to a variety of reasons. These could include (1) pathogen DNA degradation as a result of freeze–thaw cycles during sample storage, transport or inappropriate sample collection^20^, (2) low levels of bacterial DNA below the limit of detection of the Ultra in tissue homogenates, especially since no prior DNA extractions on specimens were performed^13,16^, and (3) interference associated with the composition of the tissue specimens i.e. fat composition or presence of DNA degrading enzymes^21^. However, the Ultra platform does include an in-cartridge sample probe check that will identify the presence of any qPCR inhibition prior to commencing testing^18^.

The additional MTBC DNA detection of five culture negative rhinoceros lymph node samples, seven rhinoceros culture negative BALFs, one elephant culture negative lung specimen and one elephant culture negative TW specimen by the Ultra may have been due to false negative culture results^10^, or the detection of MTBC complex DNA from dead non-viable mycobacteria in the sample, as frequently observed with specimens from human TB patients^22,23^. However, detection in tissue and respiratory samples may also represent early MTBC infection considering that the animals included in this study were all from known MTBC-endemic areas with varying MTBC prevalence’s in various wildlife species^5,7,24,25^. It is also possible that there was recognition by the Ultra of environmental nontuberculous mycobacteria (NTMs). Further comparisons to culture-based methods may help to elucidate this.

Limitations of this study include limited sample size, multiple freeze-thawing of samples prior to testing, a possible underestimated sensitivity and overestimated specificity for the assay when using animal samples. Further investigations should include the thorough evaluation of the sensitivity and specificity of the Ultra assay in animal samples. This can be achieved by testing fresh samples from more animals sampled from both MTBC endemic and negative wildlife parks with the Xpert machine as close to the animal as possible.

In conclusion, the Ultra assay appears to be a rapid diagnostic test for the detection of MTBC DNA from tissue and respiratory tract samples collected from African elephants and rhinoceros. Furthermore, the Ultra assay could provide a new tool for the detection of MTBC in various sample types from other wildlife species.

## Materials and methods

### Animals

During 2018 and 2019, 31 antemortem BALF samples were opportunistically collected from 30 free-ranging white rhinoceros and necropsy tissue samples from six poached rhinoceros (including BALF samples from rhinoceros) in KNP which is endemic for *M. bovis*. Trunk wash (TW) and BALF samples were collected from 16 African elephants that included 15 free-ranging exposed animals in KNP and one zoo elephant from Pretoria (South Africa) suspected of being MTBC-infected. In addition, tissue samples were collected from a euthanized zoo elephant and from two elephant carcasses found in KNP. Ethical approval for this project was granted by Stellenbosch University Animal Care and Use Committee (ACU‐2018‐0966; ACU‐2019‐6308 and ACUACU-2019-9081) and section 20 research permits issued by the Department of Agriculture, Forestry and Fisheries (Ref: 12/11/1/7/2; 12/11/1/7/5 and 12/11/1/7/6). Moreover, ethical approval was also granted by South African National Park (SANParks) Animal Care and Use Committee (Ref: 011/19) and all animal were handled by SANParks Veterinary Wildlife Services’ veterinarians and staff according to their guidelines.

### Specimen collection and mycobacterial culture

All animal carcasses included in this study were examined for gross bTB lesions. Tissue samples with bTB-consistent lesions were collected and stored at − 20 °C. For animals with no visible lesions, samples of head and thoracic lymph nodes were pooled by anatomical site. For antemortem samples, endoscopic BALF samples were collected endoscopically as previously described^26,27^. Additionally, TW samples were obtained from immobilized elephants by flushing 250 ml of sterile saline into each nostril, separately, elevating and lowering the trunk for approximately 30–40 s and then aspirating the fluid into a sterile 500 ml collection chamber. All respiratory samples (BALF and TW) collected in 50 ml sterile falcon tubes were concentrated by centrifugation at 2,000 ×*g* for 30 min and decanting 46 ml of the supernatant. Pellets were resuspended and the remaining 4 ml were stored at − 20 °C. All tissue and respiratory samples were processed for mycobacterial culture using Mycobacteria Growth Indicator Tubes (MGIT) and the BACTEC MGIT 960 Mycobacterial detection system (Becton Dickinson, Franklin Lakes, NJ, USA), as previously described^28^. All Ziehl–Neelsen positively stained bacterial cultures were genetically speciated by polymerase chain reaction (PCR) as previously described^29^. An additional 150 ml of BALF and TW samples were collected and frozen for all animals immobilized. Any rhinoceros and elephant with a mycobacterial culture negative/Ultra assay negative BALF and TW sample was selected for downstream spiking experiments. Animals with any culture-confirmed tissue or respiratory tract sample were defined as MTBC-infected.

### Spiking of matrices with MTBC and detection by Ultra

*M. bovis* SB0121 and *M. tuberculosis* H37Rv stock cultures, with known concentrations, were provided by the Division of Molecular Biology and Human Genetics (Stellenbosch University). To increase stock volume, both isolates were cultured in separate T25 culture flasks (ThermoFisher, USA) containing 5 ml liquid Middlebrook 7H9 Broth (Merck, USA), 0.05% Tween 80 (Merck, USA), 0.2% Glycerol and 10% Middlebrook OADC growth supplement (Merck, USA) at 37 °C for 5 days. The optical density (OD) was determined and cultures diluted to an OD = 0.05 in 30 ml 7H9 culture media in T75 culture flasks. The cultures were subsequently incubated for 2 days until it reached an OD = 0.2. Serial dilutions (10^−1^–10^−6^) were prepared in a round bottom 48-well culture plate. The neat culture and three dilutions (10^−4^, 10^−5^ and 10^−6^) thereof were plated in triplicate on vented petri dishes containing 7H11 Middlebrook Agar (Merck, USA) and 5% Glycerol. Colony forming units (CFUs) were enumerated after 30 days and the final stock concentrations (CFU/ml) determined.

Prior to use for spiking, all stocks were agitated in tubes containing sterile zirconium silica beads (Merck, USA) to remove clumps of bacteria. Thereafter, the supernatant was transferred to sterile microcentrifuge tubes and centrifuged at 3,000 ×*g* for 20 min. The supernatant was discarded, and the pellet resuspended in sterile Tris–EDTA buffer (pH 7).

In order to determine the detection range of *M. bovis* and *M. tuberculosis* bacilli by the Ultra assay, respiratory tract samples from culture-negative elephants (TW and BALF) and rhinoceros (BALF) were used as the matrices. Specific working concentrations of *M. tuberculosis* and *M. bovis* were spiked into all matrices to assay concentrations and tested as a single event on the same day as previously described^16^. Briefly, concentrations ranging from 30—2 and 0 CFU/ml for *M. tuberculosis* and 1 × 10^6^—2 and 0 CFU/ml for *M. bovis*^16^ were spiked into BALF and TW samples from 6 elephants and BALF samples from 3 rhinoceros*.* The assay was performed in duplicate for each dilution. The limit of detection (LOD) was defined as the lowest number of CFU/ml which, when spiked into 1 ml of matrix, would result in 100% detection of *M. tuberculosis* and *M. bovis* by the Ultra assay in this study. Furthermore, 1 × 10^6^ CFU/ml. *M. avium* were spiked into the respiratory sample matrix, in duplicate, and tested to determine assay specificity (Fig. 1).

### Specimen preparation and testing with Ultra

All rhinoceros and elephant clinical tissue and respiratory tract samples were processed for mycobacterial culture as previously described^28,30^. Following a second thaw of the samples, they were also processed for Ultra testing according to the Xpert MTB/RIF alternate sample testing guide. Clinical specimens were directly tested using the Ultra, sample treatment and cartridge loading were done as previously described^13^. First, each sample (TW, BALF, tissue homogenate or spiked BALF and spiked TW) was directly mixed at a ratio of 2:1 with the Xpert lysis buffer containing NaOH and isopropanol (Cepheid, Sunnyvale, CA). The mixture was incubated for 15 min with occasional shaking and then loaded into the sample chamber of the cartridge for automatic processing and analysis as previously described^16^. The presence of MTBC DNA targets was confirmed using real-time signal from probes detecting the multicopy IS*6110* and IS*1081* genes. If confirmed, the Ultra uses four *rpoB* sloppy molecular beacon (SMB) probes to determine whether mutations associated with RIF resistance is present^16^. Additionally, the “trace” category was designated to identify samples containing lower number of MTBC bacilli according to the presence of the IS*6110* and/or IS*1081* molecular signals (Ct ≤ 37) in the absence of a signal from at least 3 of the *rpoB* SMBs. Automated MTBC detection and RIF susceptibility calls were performed by modified Xpert Diagnostic software (Cepheid). Results were defined as follows: (1) “MTB not detected” (assay will not proceed to high-resolution melt); (2) “MTB trace detected” but “RIF resistance indeterminate” (assay will not proceed to melt); (3) “MTB detected high/medium/low/very low” and “RIF resistance detected/not detected,” respectively. The assay allows for the identification of the presence of MTBC DNA in samples within 60 min and RIF resistant calls in less than 90 min. Full description of assay parameters are reported in Chakravorty et al*.*^16^.

### Statistical analysis

Mean bacterial concentrations detected by the Ultra were shown as a line-graphs, left y-axis as Ct values, x-axis as CFU/ml and interquartile ranges generated for all replicates (GraphPad Prism v7.0.4, GraphPad Software). All positive and negative Ultra results and mycobacterial culture results were reported as proportions of the total number of samples tested. For all animal specimens, the agreement between the Ultra and culture was calculated as Cohen’s kappa coefficient (*κ*) using the online agreement calculator on the GraphPad software website (https://graphpad.com/quickcalcs/kappa1/). All results for the Ultra assay and mycobacterial culture results for each sample tested from individual animals are provided in the Supplemental Table.

## Supplementary information

Supplementary information.
